# What Is the Comparative Physiological and Therapeutical Action of Free Phosphorus and the Phosphites?

**Published:** 1877-05

**Authors:** 


"What is the Comparative Physiological and Therapeutical Action of Free Phosphorus and the Phosphites?" An Essay to which "the Merrit H. Cash" Prize was awarded by the New York State Medical Society, 1876. By Samuel R. Percy, M. D., Professor of Materia Medica in the New York Medical College, etc.

This is a small pamphlet, in which Dr. Percy makes direct issue against the "dogmatic assertions" of Dr. J. Ashburton Thompson, as presented in a late work entitled "Free Phosphorus in Medicine." We agree with the author in the importance he attaches to the remedy, and were very much interested in the details he presents as to its *modus operandi*, and the preparations in which it is most appropriately given.

The pamphlet tells us that Thompson's main objection to administering it in certain preparations is their liability to generate acid, and thereby become particularly deleterious to the system. This the author denies, and asserts, with corroborating experiments, that the lower oxides of phosphorus are therapeutical, and not altogether toxical. While, then, Thompson prefers the free phosphorus, the author regards the lower oxides as free from the dangers pointed out by the former, and also favors the administration of the uncombined drug under certain circumstances.

This is a very interesting subject, and we shall take great interest in its further discussion.

